# Effects of Hypertension, Diabetes, and Smoking on Age and Sex Prediction from Retinal Fundus Images

**DOI:** 10.1038/s41598-020-61519-9

**Published:** 2020-03-12

**Authors:** Yong Dae Kim, Kyoung Jin Noh, Seong Jun Byun, Soochahn Lee, Tackeun Kim, Leonard Sunwoo, Kyong Joon Lee, Si-Hyuck Kang, Kyu Hyung Park, Sang Jun Park

**Affiliations:** 1 Department of Ophthalmology, Seoul National University Bundang Hospital, Seoul National University College of Medicine, Seongnam, Republic of Korea; 2 Department of Ophthalmology, Kangdong Sacred Heart Hospital, Seoul, Korea; 3 School of Electrical Engineering, Kookmin University, Seoul, Republic of Korea; 4 Department of Neurosurgery, Seoul National University Bundang Hospital, Seongnam, Republic of Korea; 5 Department of Radiology, Seoul National University Bundang Hospital, Seongnam, Republic of Korea; 6 Division of Cardiology, Department of Internal Medicine, Seoul National University Bundang Hospital, Seongnam, Republic of Korea

**Keywords:** Biomarkers, Medical research

## Abstract

Retinal fundus images are used to detect organ damage from vascular diseases (e.g. diabetes mellitus and hypertension) and screen ocular diseases. We aimed to assess convolutional neural network (CNN) models that predict age and sex from retinal fundus images in normal participants and in participants with underlying systemic vascular-altered status. In addition, we also tried to investigate clues regarding differences between normal ageing and vascular pathologic changes using the CNN models. In this study, we developed CNN age and sex prediction models using 219,302 fundus images from normal participants without hypertension, diabetes mellitus (DM), and any smoking history. The trained models were assessed in four test-sets with 24,366 images from normal participants, 40,659 images from hypertension participants, 14,189 images from DM participants, and 113,510 images from smokers. The CNN model accurately predicted age in normal participants; the correlation between predicted age and chronologic age was R^2^ = 0.92, and the mean absolute error (MAE) was 3.06 years. MAEs in test-sets with hypertension (3.46 years), DM (3.55 years), and smoking (2.65 years) were similar to that of normal participants; however, R^2^ values were relatively low (hypertension, R^2^ = 0.74; DM, R^2^ = 0.75; smoking, R^2^ = 0.86). In subgroups with participants over 60 years, the MAEs increased to above 4.0 years and the accuracies declined for all test-sets. Fundus-predicted sex demonstrated acceptable accuracy (area under curve > 0.96) in all test-sets. Retinal fundus images from participants with underlying vascular-altered conditions (hypertension, DM, or smoking) indicated similar MAEs and low coefficients of determination (R^2^) between the predicted age and chronologic age, thus suggesting that the ageing process and pathologic vascular changes exhibit different features. Our models demonstrate the most improved performance yet and provided clues to the relationship and difference between ageing and pathologic changes from underlying systemic vascular conditions. In the process of fundus change, systemic vascular diseases are thought to have a different effect from ageing. **Research in context. Evidence before this study**. The human retina and optic disc continuously change with ageing, and they share physiologic or pathologic characteristics with brain and systemic vascular status. As retinal fundus images provide high-resolution *in-vivo* images of retinal vessels and parenchyma without any invasive procedure, it has been used to screen ocular diseases and has attracted significant attention as a predictive biomarker for cerebral and systemic vascular diseases. Recently, deep neural networks have revolutionised the field of medical image analysis including retinal fundus images and shown reliable results in predicting age, sex, and presence of cardiovascular diseases. **Added value of this study**. This is the first study demonstrating how a convolutional neural network (CNN) trained using retinal fundus images from normal participants measures the age of participants with underlying vascular conditions such as hypertension, diabetes mellitus (DM), or history of smoking using a large database, SBRIA, which contains 412,026 retinal fundus images from 155,449 participants. Our results indicated that the model accurately predicted age in normal participants, while correlations (coefficient of determination, R^2^) in test-sets with hypertension, DM, and smoking were relatively low. Additionally, a subgroup analysis indicated that mean absolute errors (MAEs) increased and accuracies declined significantly in subgroups with participants over 60 years of age in both normal participants and participants with vascular-altered conditions. These results suggest that pathologic retinal vascular changes occurring in systemic vascular diseases are different form the changes in spontaneous ageing process, and the ageing process observed in retinal fundus images may saturate at age about 60 years. **Implications of all available evidence**. Based on this study and previous reports, the CNN could accurately and reliably predict age and sex using retinal fundus images. The fact that retinal changes caused by ageing and systemic vascular diseases occur differently motivates one to understand the retina deeper. Deep learning-based fundus image reading may be a more useful and beneficial tool for screening and diagnosing systemic and ocular diseases after further development.

## Introduction

Changes occur naturally in human retina and optic disc over the lifetime in a human^1–6^. The retina and optic disc, in addition to the final structures in itself in a visual system, share some physiological characteristics with the brain as they differentiate from diencephalon during embryonic development^7^. Therefore, the effect of normal ageing changes have been studied through histological or multimodal imaging techniques^8^. Age is the single most reliable surrogate as the milestone in ageing and growing; therefore, researchers have attempted to predict patients’ chronologic age from their medical examinations and images (e.g., X-rays, facial photographs, and DNA), aiming to reflect the status of a target organ and/or the whole body^9–14^. In particular, as retinal fundus images provide high-resolution *in-vivo* images of retinal vessels and parenchyma without any invasive procedure^15^, retinal fundus images have been used to detect target organ damage in vascular diseases (e.g., hypertension and diabetes mellitus [DM]), to screen retinal and optic disc diseases (e.g., age-related macular degeneration and glaucoma), and to predict cerebral/cardiovascular diseases^16–21^. Recently, deep neural networks have revolutionised the field of medical image analysis including retinal fundus images; deep-learning algorithms presented discriminative performances comparable to those of an ophthalmologist in diagnosing diabetic retinopathy, age-related macular degeneration, and glaucoma, as well as in predicting age, sex, and presence of cardiovascular diseases^22–26^. Previous study reported highly accurate results of a mean absolute error of 3.26 years for age prediction and receiver operating characteristic curve (AUC) of 0.97 for gender prediction^25^, however, there were several limitations including a low proportion of Asian, a lack of analysis of the differences in accuracy with age, and a lack of discussion of which parts of the fundus image were used for age prediction. Virtually nothing is known regarding how a neural network predicts age and sex through fundus images, and which compartments of retinal fundus images are crucial for discrimination and identification. In addition, many systemic diseases are known to be associated with retinal diseases, and studies on the retina changes in patients with systemic diseases are actively conducted. However, how systemic diseases change retina and optic nerves and how these changes differ from age-related changes are still unclear. That is, the common features and differences between pathologic changes in and the ageing process of the retinal fundus are not well known^16^.

The convolutional neural network (CNN) proposed by Krizhevsky *et al*.^27^ demonstrated high performance in image recognition, many models for medical image recognition tasks have been developed and showed good results^28,29^. We expected the CNN to find the proper feature for the task from the retinal fundus images and to produce accurate results. We investigated how well a CNN predicts age and sex through retinal fundus images, and subsequently investigated according to the participants’ age, sex, smoking status, as well as whether the presence of hypertension and DM. Furthermore, we investigated the role of retinal vessels in predicting age and sex by inpainting vessels from retinal fundus images.

## Materials and Methods

### Dataset Organisation

We used the retinal fundus images from the Seoul National University Bundang Hospital Retina Image Archive (SBRIA) after de-identification except the age, sex, and underlying diseases at the study date; details are described in our previous study^26,30^. We included 412,026 retinal fundus images from 155,449 participants obtained at the health promotion centre in Seoul National University Bundang Hospital (SNUBH) between June 1st, 2003, and June 30th, 2016, in which detailed information regarding the presence of hypertension, DM, and the smoking status was presented. Among them, 243,668 images from normal participants without hypertension, DM, and smoking history, 40,659 images from participants with hypertension, 14,189 images from participants with DM, and 113,510 images from participants with smoking exist. The number of participants and images in each group, and the number of participants and images with overlapping diseases are shown in the Supplementary Table S1. In addition, the number and percentage of retinal fundus images according to the age and sex of each test-set are shown in Supplementary Table S2. As the participants underwent fundus photographs at least 1-year or more intervals, the fundus photographs of the same patient captured on different dates were considered to be distinct from each for age prediction. Retinal fundus images were acquired using various fundus cameras (Kowa VX-10, Kowa VX-10a, Kowa nonmyd7, Canon CF60Uvi, Canon CR6-45NM). This study was approved by the Institutional Review Board (IRB) of the SNUBH (IRB no. B-1703-386-103), and the requirement of informed consent was waived from the IRB. The study complied with the guidelines of the Declaration of Helsinki.

### Pre-processing and experimental setup

Every RGB channel of each input image was normalised to a z-score^31^. This ensures the classification results to be invariant of intensities and colour contrasts of the images, thereby enabling the model to make predictions solely based on the shape configurations of the fundi. The black background of the fundus images was excluded in normalisation.

**Figure 1:**
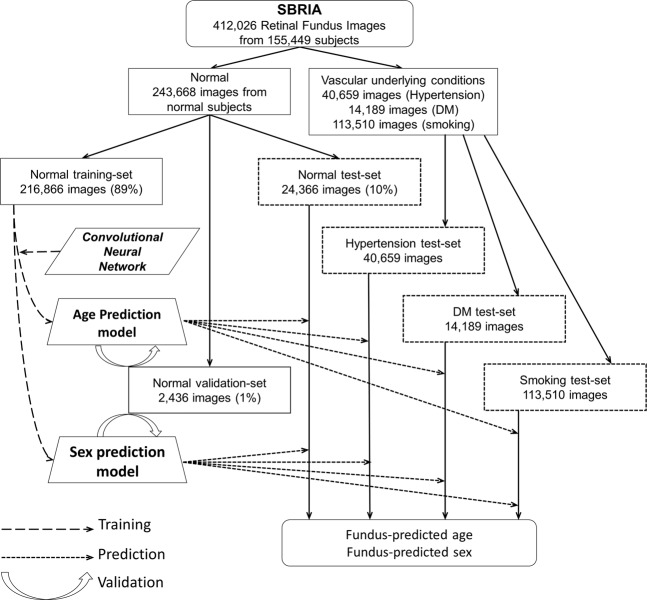
Flow chart illustrating the processes of data partitioning, training, validation, and testing. SBRIA, Seoul National University Bundang Hospital Retina Image Archive; DM, diabetes mellitus.

The images from normal participants are distributed randomly into training-set, validation-set, and test-set of ratios 89% (216,866), 1% (2,436), and 10% (24,366) by uniform sampling, respectively. We developed and trained CNN for the prediction of age and sex using the training-set from normal participants and verified the models using the validation-set while training the models. We assessed the performance of the models using the test-set of 24,366 normal images (normal test-set) and the groups of images from participants having hypertension, DM, and smoking history, separately. By performing the same number of every epoch time, we obtained the sample mean of the resulting prediction probabilities. Figure 1 shows a flow chart for the experimental setup.

### Convolutional neural network (CNN) as regressor and classifier

**Figure 2:**
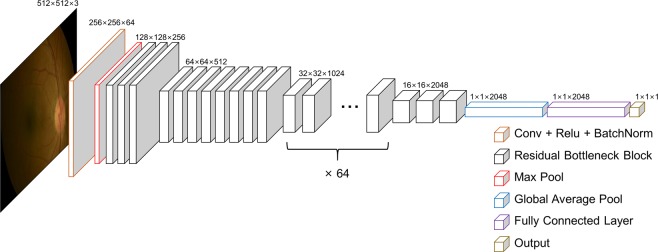
Schematics of convolutional neural network. The retinal image is an anonymous image.

Separate CNNs were trained for age and sex prediction, respectively. We used Pytorch (ver. 0.4.1, https://pytorch.org/) as the deep-learning library to implement the software to train, validate, and test the CNN. The network structure of the CNN adopted the structure of the residual network (ResNet), which had previously demonstrated high performance in a classification task^32^. ResNet has five versions (ResNet-18, 34, 50, 101, 152) depending on the depth of the convolution. We chose ResNet-152 because the deeper the convolution, the better performance. ResNet-152 has a total of 152 layers and its configuration is as follows. The set consists of a convolution layer, batch normalization, and ReLU (activation function) with 151 layers, and a fully connected last layer. Only the first convolution layer is set to 7×7 kernel with stride 2 and padding 3, and the kernel size of all subsequent convolution layers is 3×3. In addition, by setting stride 2 in 4 convolution layers, multi-scale can be considered without additional pooling layer. We used ResNet-152 as a backbone network, which was pre-trained in general image classification from ImageNet database. We used the parameters of the convolution layers in pre-trained network as our initial values, except for the fully connected last layer. The pre-trained network had learned various features through the huge amount of images from the ImageNet database. Therefore, the transfer learning method from the pre-trained network is faster than the scratched method and shows better performance^33^. While the overall structure is similar, some details were modified regarding the average pooling layer (changed kernel size from 7 to 16) and the fully connected layer (changed output dimension from 1000 to 1) to tailor the CNN to our data and desired output (Fig. 2). We define the unprocessed numerical output of the CNN as the predicted age, making the CNN for age prediction a direct regressor, while the output of the sigmoid function for the numerical output of the CNN is defined as the probability of predicted sex, making the CNN for sex prediction a classifier. As a loss function for training and error calculation, the smooth L1 loss function was adopted in the age prediction model because it has the appropriate properties of both regular features of L1 and L2^34–36^, and the binary cross-entropy function was adopted in the sex prediction models for binary classification. In addition, Adam was used as the optimization scheme (learning rate = 1e^−5^, beta1 = 0.9, and beta2 = 0.999)^37^. One epoch is defined as performing backpropagation once for all images in the total training-set, and both CNNs for age and sex prediction were learned through 10 epochs. The CNNs were verified for each epoch using the validation-set; finally, the best results were built into the prediction model.

### Class activation map analysis

To identify the region used in CNN, we used the class activation map (CAM) technique to highlight the core regions that the network focuses on for the age and sex prediction via Bayesian approximation for estimation of uncertainty in predictions^31,38,39^.

### Prediction of age and sex in retinal fundus images after inpainting retinal blood vessels

To determine the effect of retinal blood vessels in retinal fundus images on predicting age and sex, we created vessel-erased images. First, the blood vessel region was extracted using the scale-space approximated CNN (SSANet) that was previously reported by our group and demonstrated state of the art performance in retinal vessel segmentation^40^. Subsequently, we used the inpainting technique, which naturally fills holes or some regions in the image by extrapolation from the surrounding background. We applied the method previously reported by Telea *et al*.^41^, to inpaint the vascular regions, that is, to essentially erase the vessels from the retinal image. All images of the training-set, validation-set, and test-set were first pre-processed identically to the original training-set, then reconstructed as stated above, and our age and sex prediction models were trained, validated, and tested again in the same manner described above.

### Statistical analysis

To analyse the relationship between predicted age and chronologic age, the absolute values of error and their distributions were obtained from each test-set including normal, hypertension, DM, and smoking status. To further assess the statistical significance of the performance of our age prediction model, we used linear regression to obtain their coefficients of determination (R^2^) and 95% confidence interval (CI). The mean absolute error (MAE) from the chronologic age was calculated for the testing-sets, for the age subsets that are divided by decade. Additionally, to obtain the accuracy for age prediction, each result was interpreted as correct if the predicted age was concordant with the chronologic age with certain error margins of ±1, ±3, and ±5 years. The analysis of variance (ANOVA) was conducted to compare the values of squared error according to the underlying vascular conditions. Tukey’s post-test was used for pairwise group comparison. The area under a receiver operating characteristic curve (AUC) and 95% CI were used to report the performance of sex prediction. All statistical analysis was performed using Python; in particular, 95% CI were estimated by the bootstrap technique^42^.

## Results

Table 1 shows the baseline demographics of each dataset. The mean chronologic age (years ± standard deviation) was 46.70 ± 16.67 in the normal training-set, 46.63 ± 15.86 in the normal validation-set, and 46.64  ±  15.83 in the normal test-set. The mean chronologic age (years) in the fundus photographs from participants with underlying vascular conditions was 56.71  ±  9.66 in the hypertension test-set, 56.56  ±  9.75 in the DM test-set, and 49.35  ±  10.05 in the smoking test-set. All test-sets consisted of more males than females, especially in the smoking group (normal, male 55.1%; hypertension, male 77.4%; DM, male 74.1%; smoking, male 93.5%).

**Table 1:** Baseline demographics of normal training-set and validation set, and test-sets with various conditions: normal, hypertension, diabetes, and smoking.

	Sex	Number of subjects	Fundus photo image, N (%)	Age, mean (SD, range)
**Normal**				
Training-set	All	84,526	216,866	46.70 (16.67, 0-105)
	Male	44,714 (52.90%)	119,442 (55.08%)	46.68 (14.80, 0-105)
	Female	39,812 (47.10%)	97,424 (44.92%)	46.73 (18.70, 1-98)
Validation-set	All	2,397	2,436	46.63 (15.86, 1-92)
	Male	1,341 (55.94%)	1,372 (56.32%)	46.46 (15.18, 2-87)
	Female	1,056 (44.06%)	1,064 (43.68%)	46.84 (16.69, 1-92)
Test-set	All	20,823	24,366	46.64 (15.83, 0-95)
	Male	11,327 (54.40%)	13,427 (55.11%)	46.69 (14.90, 1-92)
	Female	9,496 (45.60%)	10,939 (44.89%)	46.57 (16.91, 0-95)
Hypertension test-set	All	12,168	40,659	56.71 (9.66, 20-90)
	Male	7,762 (63.79%)	27,389 (77.36%)	55.19 (9.65, 22-90)
	Female	4,406 (36.21%)	13,270 (32.64%)	59.83 (8.90, 20-86)
Diabetes test-set	All	4,545	14,189	56.56 (9.75, 19-90)
	Male	3,191 (70.21%)	10,509 (74.06%)	55.49 (9.40, 19-90)
	Female	1,354 (29.79%)	36,80 (25.94%)	59.63 (10.06, 23-86)
Smoking test-set	All	30,990	113,510	49.35 (10.05, 18-86)
	Male	28,305 (91.34%)	106,116 (93.49%)	49.69 (9.92, 18-86)
	Female	2,685 (8.66%)	7,394 (6.51%)	44.39 (10.64, 18-82)

Data are expressed by means and standard deviations.

**Figure 3:**
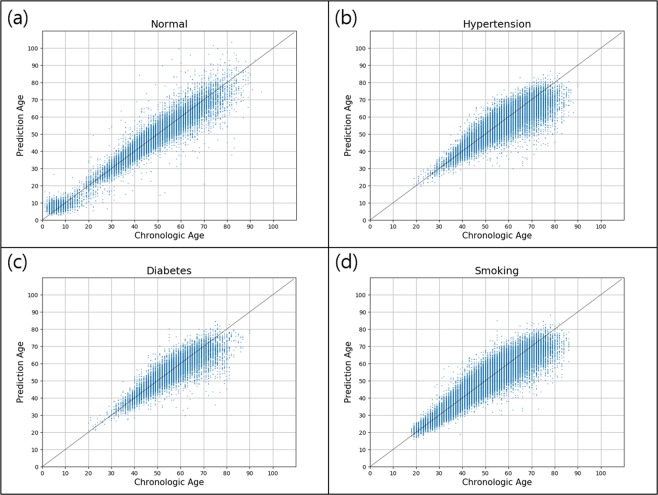
Predicted and chronologic ages in the four test-sets: (**a**) normal, (**b**) hypertension, (**c**) diabetes, (**d**) smoking. The lines represent y = x values. Fair linear relationships were indicated in normal test-set (R^2^ = 0.92, 95% confidence interval [CI] 0.92-0.93), while the others indicated relatively loose relationship as follows: hypertension, R^2^ = 0.75, 95% CI 0.75–0.76; diabetes mellitus, R^2^ = 0.75, 95% CI 0.74–0.75; smoking, R^2^ = 0.86, 95% CI 0.86–0.86.

The normal test-set of 24,366 images indicated a MAE of 3.06 (95% CI, 3.03-3.09) years. The MAE increased significantly in the hypertension test-set (3.46 [95% CI, 3.44-3.49] years) and DM test-set (3.55 [95% CI, 3.50-3.60] years), while it decreased in the smoking test-set (2.76 [95% CI, 2.75-2.77] years). Figure 3 presents a scatter plot of the linear relationship between chronologic age and predicted age in each test-set. Supplementary Fig. S1 presents a box plot of the squared error values ([predicted age – chronologic age]^2^) of the four groups. ANOVA showed significant difference at the P < 0.05 level for the squared error values of the four underlying vascular conditions, and post hoc comparisons also indicated that the means of squared error values for each vascular condition were different. (P < 0.05, Supplementary Table S3). The normal test-set demonstrated a fairly linear relationship (R^2^ = 0.92 [95% CI, 0.92-0.93]), and other test-sets demonstrated relatively low coefficients of determination: R^2^ of 0.74 (95% CI, 0.75-0.76), 0.75 (95% CI, 0.74-0.75), and 0.86 (95% CI, 0.86-0.86) in hypertension, DM, and smoking test-sets, respectively. Based on the assumption that age prediction was correct when the predicted and chronologic ages differed by an error margin of ±5 years, we observed an 82.8% correct prediction in the normal test-set. The accuracies of prediction were 77.6%, 77.0%, and 85.6% in the hypertension, DM, and smoking test-set, respectively. The accuracy for the prediction of age within ±1 year was less than 30% in all groups, and that within ±3 years was more than 55% in all groups (Table 2).

**Table 2:** Mean absolute error (MAE) and its 95% confidence interval (CI); coefficient of determination (R^2^) and its 95% CI; accuracy in predicting age within maximum differences of ±1, ±3, and ±5 years in each test-set.

Test-set	Mean Absolute Error, year (95% CI)	Coefficient of determination, R^2^ (95% CI)	Accuracy for age prediction by error margin		
			±1 year	±3 years	±5 years
Normal	3.06 (3.03-3.09)	0.92 (0.92-0.93)	24.00%	62.11%	82.82%
Hypertension	3.46 (3.44-3.49)	0.75 (0.75-0.76)	20.78%	56.33%	77.63%
Diabetes	3.55 (3.50, 3.60)	0.75 (0.74-0.75)	20.42%	55.09%	77.01%
Smoking	2.76 (2.75, 2.77)	0.86 (0.86-0.86)	25.25%	64.79%	85.59%

CI, Confidence interval.

**Figure 4:**
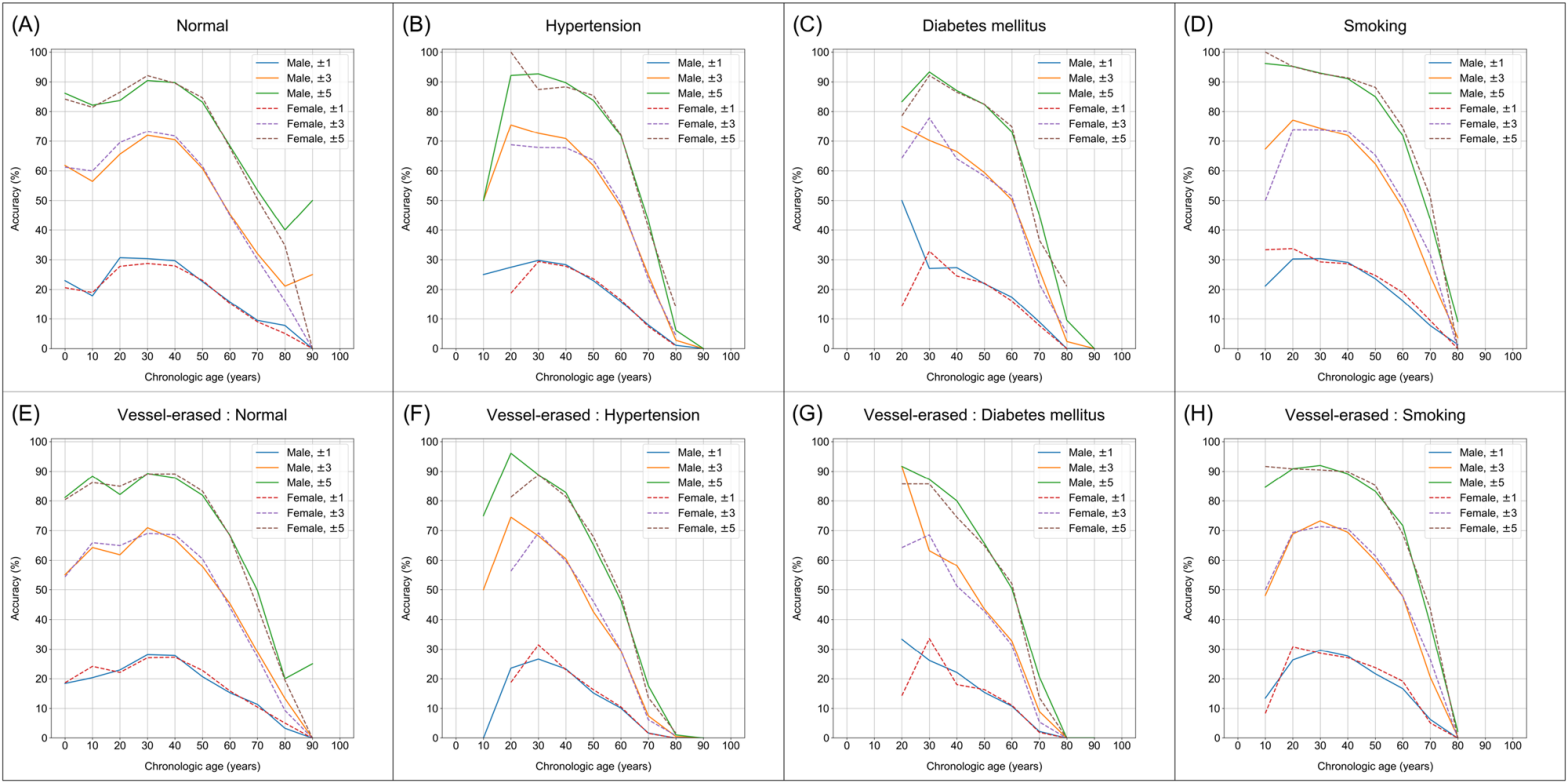
Changes in accuracy of age prediction according to chronologic age subgroups (divided by 10 years) by error margins of ±1, ±3, and ±5 years in the four test-sets and inpainted vessel-erased images from all test-sets: (**A**) normal, (**B**) hypertension, (**C**) diabetes, (**D**) smoking, (**E**) inpainted normal images, (**F**) inpainted hypertension images, (**G**) inpainted diabetes images, (**H**) inpainted smoking images. The accuracies were the highest in the 20 s to 40 s in all test-sets, decline gradually with age, and decrease significantly after 60 years old. Differences according to sex were not obvious. In inpainted vessel-erased images, the accuracy was similar compared with original images in all test-sets.

**Figure 5:**
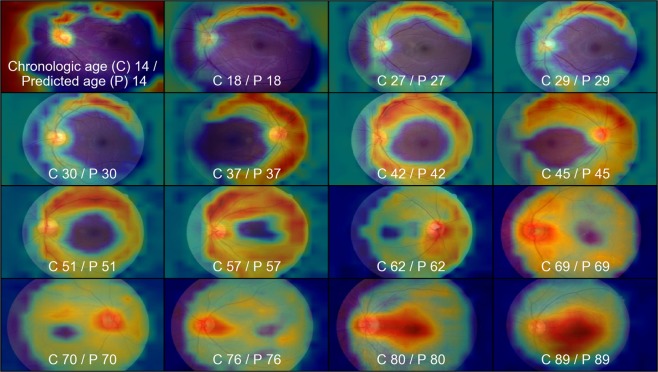
Representative class activation mapping (CAM) heat-map and its original image in age prediction model. It shows the regions that have higher influence on the prediction results in red, relative to the regions with lower influence, in blue. The CAM of the age prediction model indicated activation primarily in the vascular region.

When each test-set was divided into age subgroups by decades, the MAEs of the estimation in age groups between 20 and 59 years were below 3.0 years in all test-sets. Table 3 shows a fairly consistent MAE and good accuracy under 60 years old; however, an increased MAE and declined accuracy were exhibited in groups aged above 60 years old. Table 4 shows the MAEs and accuracies of the estimations according to three age categories (1: <30 years; 2: 30–59 years; 3: ≥60 years) in each test-set. The MAE in category 3 were higher than that in category 2 in all test-sets (normal, 4.96 vs. 2.60; hypertension, 4.75 vs. 2.69; DM, 4.74 vs. 2.84; smoking, 4.42 vs. 2.47). The accuracies for age prediction within ±5 years were more than 80% in categories 1 and 2, but less than 70% in category 3. Figure 4 shows the changes in accuracy of age prediction according to chronologic age subgroups (divided by 10 years) by error margins of ±1, ±3, and ±5 years in the four test-sets. The accuracy was the highest at 30–40 years of age in the normal and DM test-sets, and 20–30 years in the hypertension and smoking test-sets. The accuracies of age prediction declined gradually with increasing age and deteriorated after 60 years old. The accuracies of the age prediction did not differ according to sex in all groups. Representative images of the CAM heat-map in our age prediction model are shown in Fig. 5. It shows the regions that have higher influence on the prediction results in red, relative to the regions with lower influence, in blue. The CAM of our age prediction model indicated activation primarily in the vascular region.

**Table 3:** Mean predicted age, mean absolute error (MAE) and its 95% confidence interval (CI); accuracy for predicting age within maximum differences of ±1, ±3, and ±5 years, respectively, in each test-set divided by 10-year age subgroup.

Test-set	Age group	Fundus photo images, N	Mean chronologic age, year	Mean predicted age, year	Mean absolute error, year (95% CI)	Accuracy for age prediction by error margin		
						±1 year	±3 years	±5 years
Normal	0-9	1,345	5.72	8.09	2.82 (2.70-2.95)	21.71%	61.49%	85.13%
	10-19	473	13.30	11.51	3.02 (2.86-3.19)	18.39%	58.14%	81.82%
	20-29	746	25.56	25.57	2.82 (2.64-3.01)	29.22%	67.56%	85.12%
	30-39	3,317	35.67	36.01	2.35 (2.29-2.42)	29.57%	72.63%	91.23%
	40-49	7,681	44.61	44.83	2.40 (2.36-2.45)	28.97%	70.95%	89.68%
	50-59	6,360	53.92	53.46	2.97 (2.92-3.04)	22.81%	61.08%	83.82%
	60-69	3,061	63.96	62.06	4.29 (5.18-4.40)	15.52%	45.18%	68.02%
	70-79	1,169	73.47	69.82	6.08 (5.83-6.32)	9.24%	30.97%	51.84%
	80-89	208	82.84	76.57	8.45 (7.68-9.25)	6.25%	18.27%	37.02%
	90-99	6	91.33	82.20	9.51	0.00%	16.67%	33.33%
Hypertension	0-9	0	—	—	—	—	—	—
	10-19	4	19.00	22.22	3.22	25.00%	50.00%	50.00%
	20-29	118	26.66	27.77	2.14 (1.91-2.38)	26.27%	74.58%	93.22%
	30-39	1,181	36.41	37.02	2.30 (2.20-2.40)	29.72%	72.06%	92.04%
	40-49	7,959	45.46	46.08	2.43 (2.39-2.46)	28.22%	70.29%	89.45%
	50-59	16,064	54.56	54.36	2.85 (2.81-2.88)	23.13%	62.28%	84.28%
	60-69	11,226	64.02	61.62	3.85 (3.80-3.90)	16.15%	48.41%	71.97%
	70-79	3,811	73.01	66.78	6.79 (6.66-6.92)	7.69%	24.17%	42.04%
	80-89	294	81.81	69.27	12.61 (12.03-13.25)	1.02%	3.40%	9.18%
	90-99	2	90.00	78.34	11.65	0.00%	0.00%	0.00%
Diabetes	0-9	0	—	—	—	—	—	—
	10-19	0	—	—	—	—	—	—
	20-29	26	25.00	26.93	2.38 (1.17-3.10)	30.77%	69.23%	80.77%
	30-39	469	36.48	37.04	2.26 (2.13-2.40)	28.78%	72.49%	92.96%
	40-49	2,947	45.51	46.18	2.64 (2.57-2.71)	26.94%	66.14%	86.97%
	50-59	5,449	54.63	54.66	3.00 (2.95-3.06)	21.95%	59.17%	82.42%
	60-69	3,869	63.98	62.02	3.75 (3.67-3.83)	16.90%	50.66%	73.79%
	70-79	1,305	73.02	66.69	6.98 (6.74-7.22)	8.51%	24.67%	41.76%
	80-89	122	81.51	69.63	11.90 (10.94-12.89)	0.00%	3.28%	13.11%
	90-99	2	90.00	78.34	11.65	0.00%	0.00%	0.00%
Smoking	0-9	0	—	—	—	—	—	—
	10-19	64	18.84	20.89	2.34 (2.03-2.66)	23.44%	64.06%	96.88%
	20-29	2,345	26.33	26.86	2.05 (2.00-2.11)	31.00%	76.42%	95.18%
	30-39	14,960	35.86	36.11	2.16 (2.14-2.19)	30.20%	74.29%	92.91%
	40-49	41,915	44.74	44.95	2.30 (2.28-2.31)	29.07%	71.98%	91.07%
	50-59	36,778	53.86	53.40	2.78 (2.76-2.80)	23.63%	62.44%	85.14%
	60-69	13,887	63.70	61.24	3.80 (3.76-3.94)	16.28%	47.74%	71.92%
	70-79	3,340	72.86	66.98	6.49 (6.36-6.63)	7.78%	24.85%	43.86%
	80-89	221	81.61	69.43	12.21 (11.56-12.87)	1.36%	3.62%	9.05%
	90-99	0	—	—	—	—	—	—

Consistent MAE and fair accuracy are shown under 60 years old; however, increased MAE and declined accuracy are shown in age subgroup above 60 years old.

CI, Confidence interval.

**Table 4:** Mean predicted age, mean absolute error (MAE) and its 95% confidence interval (CI); accuracy in predicting age within maximum differences of ±1, ±3, and ±5 years, divided into three categories in each test-set as follows: (1) age under 30; (2) age 30–59; (3) age over 60.

Test-set	Age group	Fundus photo images, N	Mean chronologic age, year	Mean predicted age, year	Mean absolute error, year (95% CI)	Accuracy for age prediction by error margin		
						±1 year	±3 years	±5 years
Normal	0–29	2,564	12.89	13.81	2.86 (2.77–2.95)	23.28%	62.64%	84.52%
	30–59	17,358	46.31	46.31	2.60 (2.57–2.63)	26.83%	67.66%	87.83%
	60–99	4,444	67.38	64.81	4.96 (4.85–5.07)	13.41%	40.14%	62.26%
Hypertension	0–29	122	26.41	27.59	2.18 (1.94–2.42)	26.23%	73.77%	91.80%
	30–59	2,5204	50.83	50.93	2.69 (2.66–2.71)	25.04%	65.26%	86.28%
	60–99	15,333	66.60	63.05	4.75 (4.70–4.81)	13.75%	41.52%	63.31%
Diabetes	0–29	26	25.00	26.93	2.38 (1.71–3.10)	30.77%	69.23%	80.77%
	30–59	8,865	50.64	50.91	2.84 (2.80–2.89)	23.97%	62.19%	84.49%
	60–99	5,298	66.62	63.35	4.74 (4.64–4.84)	14.44%	43.15%	64.48%
Smoking	0–29	2,409	26.14	26.71	2.06 (2.01–2.12)	30.80%	76.09%	95.23%
	30–59	93,653	46.90	46.86	2.47 (2.45–2.48)	27.11%	68.60%	89.03%
	60–99	17,448	65.68	62.44	4.42 (4.38–4.47)	14.47%	42.80%	65.75%

A small MAE is shown under 3.0 (years) in categories 1 and 2, and a large MAE over 4.0 (years) in category 3. Accuracy for predicting age within ±5 years was higher than 80% in categories 1 and 2, but less than 70% in category 3.

CI, Confidence interval.

**Figure 6:**
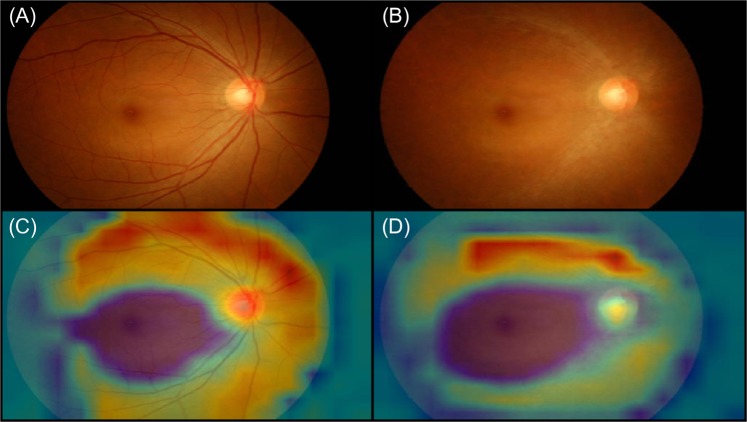
Class activation mapping (CAM) heat-map in original fundus photograph image and retinal vessel-erased image in age prediction model. CAM heat-map still focused on the retinal vascular arcade area, thus resulting in an obvious decline in the accuracy of age prediction, as shown in Table 5.

The age prediction model trained by the vessel-erased images was assessed to obtain the MAE and accuracy in the vessel-erased images in all test-sets. The predicted age from the vessel-erased images indicated a similar MAE of 3.19 years (Table 5), and also showed similar changes with age (Fig. 4). In the retinal vessel-erased images, attention is still focused on the area where blood vessels are present (Fig. 6).

**Table 5:** Analysis of reconstructed retinal vessel-erased images - mean predicted age, mean absolute error (MAE) and its 95% confidence interval (CI); accuracy for predicting age within maximum differences of ±1, ±3, and ±5 years, divided into three categories in each test-set as follows: (1) age under 30; (2) age 30–59; (3) age over 60.

Reconstructed test-set of retinal vessel-erased images	Age group	Fundus photo images, N	Mean chronologic age, year	Mean predicted age, year	Mean absolute error, year (95% CI)	Accuracy for age prediction by error margin		
						±1 year	±3 years	±5 years
Normal	0–29	2,564	12.89	14.58	5.92 (5.77, 6.07)	20.36%	59.13%	82.76%
	30–59	17,358	46.31	46.54	6.70 (6.68, 6.73)	25.38%	64.93%	86.35%
	60–99	4,444	67.38	63.89	4.32 (4.28, 4.36)	13.77%	38.73%	60.31%
Hypertension	0–29	122	26.41	30.60	4.46 (4.11, 4.84)	5.74%	29.51%	61.48%
	30–59	25,204	50.83	52.98	3.99 (3.96, 4.03)	17.89%	48.87%	70.68%
	60–99	15,333	66.60	64.24	4.57 (4.52, 4.62)	14.86%	42.65%	64.36%
Diabetes	0–29	26	25.00	30.48	5.58 (4.54, 6.70)	0.00%	15.38%	61.54%
	30–59	8,865	50.64	52.79	4.01 (3.95, 4.07)	18.33%	48.34%	70.23%
	60–99	5,298	66.62	64.47	4.58 (4.49, 4.66)	15.16%	42.90%	64.59%
Smoking	0–29	2,409	26.14	31.74	5.92 (5.77, 6.07)	8.18%	26.90%	47.07%
	30–59	93,653	46.90	53.21	6.70 (6.68, 6.73)	9.24%	27.50%	44.91%
	60–99	17,448	65.68	66.74	4.32 (4.28, 4.36)	15.22%	43.67%	66.10%

A small MAE is shown under 3.0 (years) in categories 1 and 2, and a large MAE over 4.0 (years) in category 3. Accuracy for predicting age within ±5 years was higher than 80% in categories 1 and 2, but less than 70% in category 3.

CI, Confidence interval.

**Figure 7:**
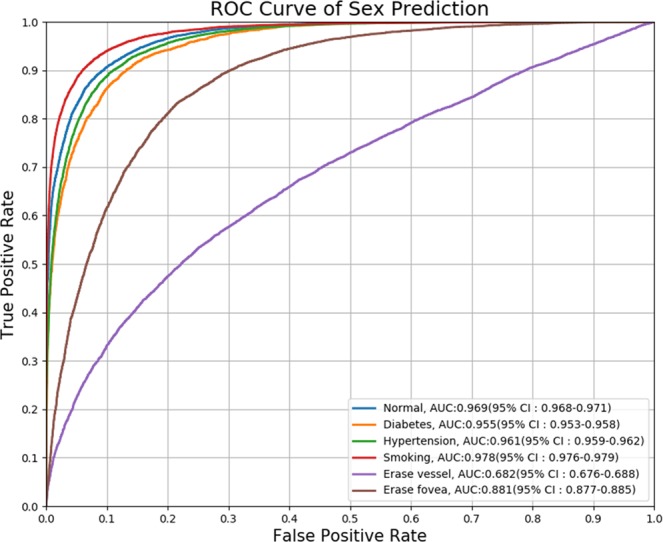
Receiver operating characteristic (ROC) curves in four test-sets and inpainting (vessel-erased and fovea-erased) images. The area under curve (AUC) was more than 0.96 in every test-set regardless of underlying condition, while the AUCs in the inpainting images were much smaller.

**Figure 8:**
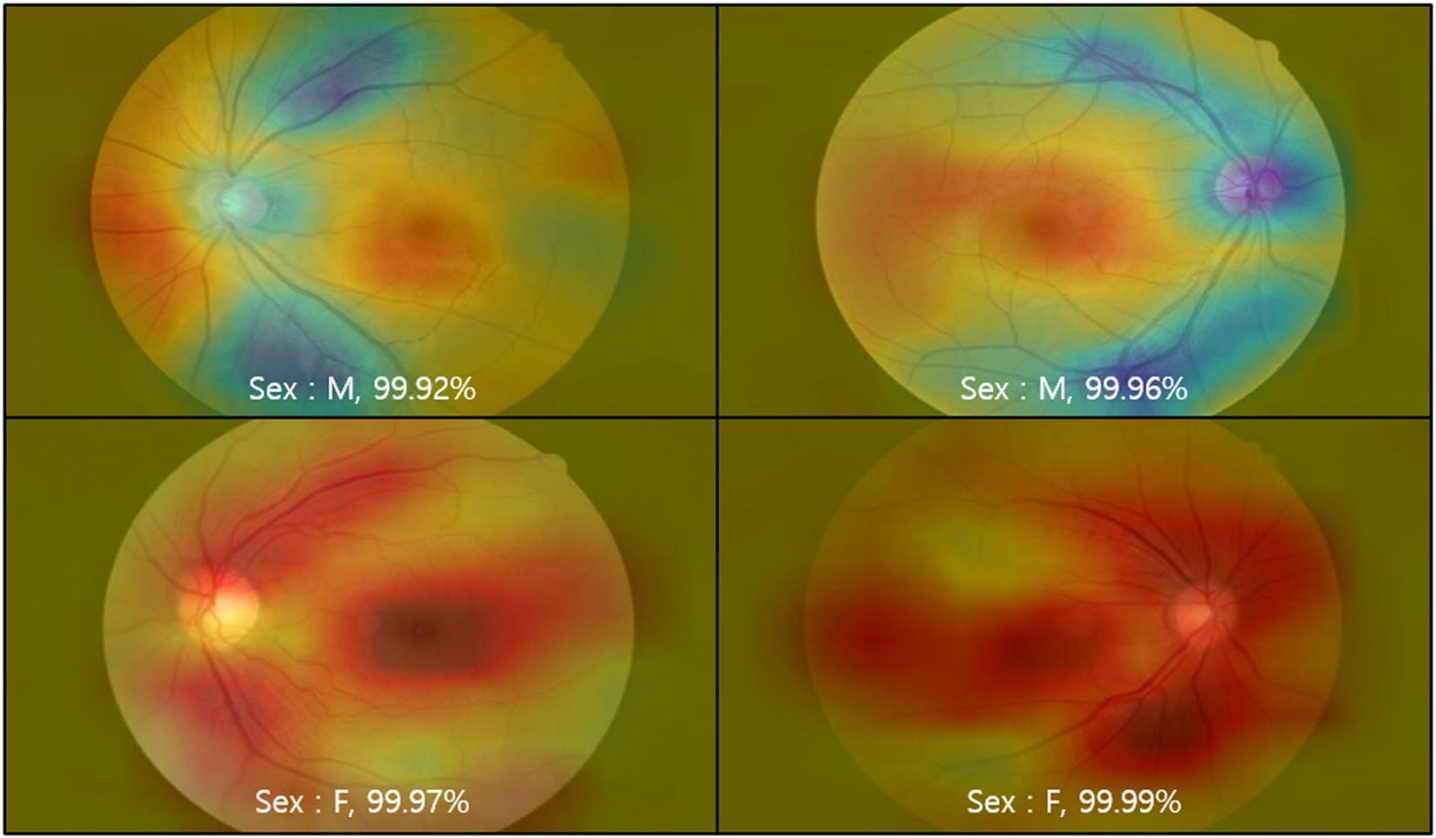
Representative class activation mapping (CAM) heat-map in sex prediction model. The area painted in red is the predominant region for sex prediction, while the blue area is used less. The CAM heat-map indicated various focuses in fovea, optic disc, and retinal vessels; in particular, proximal retinal vascular arcade lesion was prominently activated in females.

Our sex prediction model was trained and validated using the same training-set and validation-set that were tested in four test-sets; it demonstrated excellent accuracies, where the AUC was 0.97 in the normal test-set, and similar AUCs above 0.96 were shown in the other test-sets with underlying vascular conditions (hypertension, 0.96; DM, 0.96; smoking, 0.98). To confirm the significance of the fovea and retinal vessels in the prediction of sex, we generated inpainted images with erased fovea and retinal vessels, separately. The AUC was 0.881 (95% CI, 0.877–0.885) in the fovea-erased image and 0.682 (95% CI, 0.676–0.688) in the retinal vessel-erased image (Fig. 7). Representative images of the CAM heat-map in our sex prediction model are shown in Fig. 8. The CAM of our sex prediction model indicated various activations in the fovea, optic disc, and retinal vessel; in particular, the proximal vascular region was prominently activated in females.

## Discussion

Historically, researchers have sought a relevant biomarker among medical images that can reflect chronologic ageing or biological ageing. Retinal fundus images are easy to capture, inexpensive, non-invasive, and provide high-resolution images of retinal blood vessels as well as of retina and optic nerve that change according to age and vary with sex. In this respect, we developed algorithms predicting chronologic age in retinal fundus images using deep neural networks, and the algorithms demonstrated fair performance in predicting age and sex. Interestingly, the performance was the best in participants aged 20–40 years, decreased as age increased, and poor in participants aged 60 years or over. In addition, the performance deteriorated in participants suffering hypertension or DM with increasing age. The performance of the sex prediction model demonstrated high accuracy and showed no inferiority in test-sets with underlying disease.

CNN have led to a series of breakthroughs for computer vision area including image classification, semantic segmentation, object detection, and bounding box regression^27,43–45^. Network models such as VGG net, GoogLeNet, and ResNet have been widely used to date^46^. Among them, ResNet proposed the concept of residual blocks to improve performance that can be degraded in very deep networks, and solved the interference with convergence such as vanishing/exploding gradients^47–50^. Through this, ResNet showed better performance compared to VGG net and GoogLeNet in deep network learning. ResNet also demonstrated high performance for medical image recognition tasks such as retinal vessel segmentation^40^. Therefore we used ResNet, which has the advantages mentioned above, to develop an age and sex prediction model using the retinal fundus images.

Deep-learning based age and sex estimation using retinal fundus images has been previously studied by other group. Poplin *et al*. reported the predictions of age, gender, and cardiovascular risk factors using fundus photograph data from UK Biobank and EyePACS^25^. In the algorithm validation using the UK Biobank validation dataset (n = 12,026 patients), the MAE was 3.26 (95% CI, 3.22-3.31) and R^2^ = 0.74 (95% CI, 0.73-0.75). In the algorithm validation using the EyePACS-2K validation dataset (n = 999 patients), the MAE was 3.42 (95% CI, 3.23-3.61) and the R^2^ was 0.82 (95% CI, 0.79-0.84). The MAE of the age prediction validation in our study was 3.06 (95% CI, 3.03–3.09) and the R^2^ was 0.92, which demonstrated a better or comparable performance than those of previous study. Although there are differences in the data set, the present study showed comparable results using a large number of Korean’s fundus images. The cross validation of each other’s dataset may be required to confirm the superiority of accuracy.

Vascular risk-scoring methods use equations based on large cohort studies, such as the US Framingham Heart and Offspring Studies^51^, the recent European Systematic Coronary Risk Evaluation project^52^, and the German Prospective Cardiovascular Münster study^53^. The Framingham score calculation, a well-known algorithm, considers age, sex, total and HDL cholesterol, systolic blood pressure, and smoking^51^. Additionally, various novel biomarkers such as intravascular ultrasound in left main coronary arteries^54^ and the ankle brachial index^55^ have been studied to predict vascular age and risk. Retinal images can also be used as a tool for such predictions, and this application has been demonstrated in our study using fundus photograph images and the CNN algorithm. In addition, retinal vessels and cerebral vessels exhibit similarity and *in vivo* direct observations of retinal vessels provide information about cerebral vessels^56,57^. Therefore, efforts to find and understand cerebral and systemic blood vessels through retinal fundus images are worthwhile. Furthermore, retinal images will be more interesting and valuable in systemic vascular diseases such as hypertension and DM.

Our age prediction model demonstrated good accuracies in ages under 60 years; however, in ages 60 or over, the performance deteriorated significantly. This is because ageing changes observed in retinal fundus occur continuously until the age of 60 years, and may saturate at approximately 60 years of age; subsequently, after the age of 60 years, the ageing changes may not be obvious with age. The CAM images of our age prediction model demonstrated activation primarily in the vascular region; this may be another evidence to explain the focus of the deep-learning CNN, however, similar results on vessel-erased images showed that not only blood vessels were used for prediction. In other words, optic disc, papillary vessels, and retinal parenchyma can be used to predict age. Our research was inspired by the effects of ageing on the human fundus, beyond reconfirmation of the possibility or capability of age prediction by deep learning. Interestingly, our age prediction model demonstrated similar MAEs in each test-set categorised by underlying diseases, implying that pathologic changes occurring in systemic vascular diseases are different form the changes in the ageing process. In other words, our results demonstrated that systemic vascular diseases such as hypertension, DM, and smoking resulted in various inconsistent changes in the retinal blood vessels and resulted in loosened relationships (increased coefficient of determination, R^2^). In addition, statistical analysis using ANOVA and post-hoc test for values of absolute errors in each test-set show significant differences in age prediction when the underlying disease is present.

The ageing process causes many changes in the retina and optic nerve that consist of several layers including RNFL, ILM, ONL, PRL, and RPE^8,58^. Retinal vascular changes include decrease in cellularity of peripheral capillaries and diminution of the number of capillaries of the fovea^8,59^. Thickening and hyalinisation of the vessel wall, and arteriosclerotic changes may also develop in retinal vessels with ageing^8^. In addition, the density of the choriocapillaris that provides nutritional support for the RPE and outer retina, decreases with ageing^60–62^. In particular, the increase in flow deficit of the choriocapillaris is prominent in the central 1-mm circle of the macula^62^. The fundus autofluorescence from lipofuscin of RPE correlates with age, and indicates age-dependent changes in the fovea^63^. The reason for such changes is that the human body is always maintained through metabolism, and in this process, cells and tissues are damaged while by-products accumulate^2,64^. Hence, changes due to normal ageing and pathological changes share a similar feature and cannot be completely distinguished. In particular, the ageing process and hypertension are known to be associated with vascular network changes such as retinal vascular junctional bifurcation angles^65^. Therefore, the predicted age is expected to be higher than the chronologic age in the presence of hypertension. However, the MAE and difference between the predicted age and chronologic age in the hypertension test-set were similar to that of the normal test-set in our study. This suggests that changes in retinal vessels due to hypertension show some similar features to ageing, but not entirely mimicking normal ageing. DM is another chronic systemic vascular disease that causes changes in whole blood vessels including retinal vessels. The prevalence of any diabetic retinopathy in 35 population-based studies from 22,896 patients was 34.6% overall among diabetic patients^66^. Additionally, DM altered all blood vessels and affected the diameter of the retinal blood vessels; however, the branching angle was not significantly different from those of normal participants^67^. Several studies have reported that wider retinal venular diameters and narrower arteriolar diameters were associated with the presence of diabetic nephropathy and severe levels of diabetic retinopathy^68–70^. In a type-1 DM cohort study, age (odds ratio [OR] per 10 years, 2.43 and 2.02) and retinopathy severity (OR per level, 1.14 and 1.21) were associated with focal retinal arteriolar narrowing and A/V nicking, respectively^68^. Another type-1 DM cohort study indicated that both wider venular diameters and smaller arteriolar diameters were predictors of the 16-year development of nephropathy, neuropathy, and proliferative retinopathy^71^. In a type-2 DM cohort study, smaller retinal arteriolar calibers exhibited associations with increasing age and mean arterial BP, and a larger retinal venular caliber was associated with increasing severities of retinopathy and cigarette smoking^69^. Because the level of diabetic retinopathy and tissue oxygen demand affect the changes in blood vessels^69,72^, retinal vessels in diabetic patients show various changes, thus resulting in a smaller R^2^ (coefficient of determination) in diabetic participants than normal participants. In other words, pathologic retinal changes result from various factors; our study suggests that ageing and pathological changes are not exactly the same. Unlike the other groups, the smoking group showed better performance on age prediction. There are several studies on the effects of smoking on retinal vessels^73,74^, but this does not explain how they perform better than normal. Moreover, there are spectral domain optical coherence tomography studies which shows the thickness of retinal layers in healthy chronic smokers was not significantly different to those of healthy individuals^75,76^. Sex, age, and confounding factors were likely to improve predictions, in other words, a large number of men over 30 years of age were included in our study, resulting in uniform results and higher R^2^ values.

Our prediction model predicted sex with good accuracy in all test-sets unlike the results of age prediction. A previous study indicated that the AUC of UK Biobank was 0.97 (95% CI, 0.966-0.971) and that of EyePACS-2K was 0.97 (95% CI, 0.96-0.98), which are comparable to our study^25^. Considering that the prediction accuracy is reduced significantly in the fovea-erased images and the accuracy is reduced significantly in the vessel-erased images, both the fovea and blood vessel are used for sex prediction where the blood vessel is the core region. Interestingly, the presence of underlying vascular conditions did not indicate a significant effect on sex prediction. This indicates that alterations in retinal blood vessels due to underlying vascular conditions did not exceed beyond sexual differences. Unexpectedly, our sex prediction model showed the highest AUC in the smoking group. This is probably due to the fact that the sex ratio of smokers is biased towards men (Supplementary Table S2). Sex prediction has been studied in forensic science typically, using bones or bone fragments to produce estimating formulas^77–79^. However, these human structures are expected to exhibit significant differences between sexes, and they can be determined or measured by human examiners without a computer. Considering that sex identification in retinal fundus images proved to be almost impossible even when an inspection was performed by experienced ophthalmologists, our results suggest that deep-running is superior to human perception in image discrimination and identification.

Some limitations were present in this study. First, the type of underlying disease and the duration of the disease were not considered, and images showing any ocular disease were not included. Next, changes in retinal fundus images caused by lens yellowing, cataract, and cataract surgery might affect age prediction^80^; however, the consideration of lens status is lacking in this study. Subsequently, myopia and axial length could cause significant changes in the fundus and optic nerve^81,82^. It was reported that an increased axial length was associated with arteriolar and venular narrowing; however, the arteriovenous diameter ratio or vessel junctions were not affected significantly by the axial length^81^. It is unclear which components of the retinal vasculature were used to predict age and sex; at the least, the effects of arteriovenous diameter ratio and junctional exponents were considered to be less disturbed by the axial length^81^. Next, confounding factors may appear depending on the fundus photograph camera model or its manufacturer. Differences in image size and telecentricity were reported depending on the fundus imaging system^83^. However, the absolute size of the targets by each fundus imaging system may be different; nevertheless, the ratio of each target size may not be significant. Finally, our study has not been validated in other databases and only Koreans are included in the study. Differences in retina according to ethnicity have been reported^84,85^, therefore, further validation studies involving other database especially other ethnicities are warranted.

Our model demonstrates accurate and highly reliable age estimates especially in normal participants under age 60 years. Retinal fundus images from participants with underlying conditions (hypertension, DM, or smoking) indicated relatively low coefficients of determination (R^2^) between the predicted age and chronologic age, thus suggesting that the ageing process and pathologic vascular changes exhibit different features. Fundus-predicted sex indicated an accuracy of 0.96 of AUC score in all groups. Our CNN-based age and sex prediction model has demonstrated the most improved performance to date. Our research suggests that ageing and systemic vascular diseases have different effects on the retina. Further research on the clinical significance and application of our model to other population groups is needed.

## Supplementary information

Supplementary Information.

## Data Availability

The datasets generated during and/or analysed during the current study are available from the corresponding author on reasonable request.
