# Supplementary material for: Effects of Hypertension, Diabetes, and Smoking on Age and Sex Prediction from Retinal Fundus Images
Source: Sci Rep. 2020 Mar 12;10:4623. doi: 10.1038/s41598-020-61519-9 (PMC7067849; doi:10.1038/s41598-020-61519-9)
Supplement: Supplementary file 1 — Supplementary Information. [file 41598_2020_61519_MOESM1_ESM.pdf]

# Effects of Hypertension, Diabetes, and Smoking on Age and Sex Prediction from Retinal Fundus Images

Yong Dae Kim, MD[1,2]*; Kyoung Jin Noh, MS[1]*; Seong Jun Byun, MS[1]*; Soochahn Lee, PhD[3]; Tackeun Kim, MD[4]; Leonard Sunwoo, MD[5]; Kyong Joon Lee, PhD[5]; Si-Hyuck Kang, MD[6]; Kyu Hyung Park, MD, PhD[1]; Sang Jun Park, MD, MSc[1]

[1]Department of Ophthalmology, Seoul National University Bundang Hospital, Seoul National University College of Medicine, Seongnam, Republic of Korea

[2]Department of Ophthalmology, Kangdong Sacred Heart Hospital, Seoul, Korea

[3]School of Electrical Engineering, Kookmin University, Seoul, Republic of Korea

[4]Department of Neurosurgery, Seoul National University Bundang Hospital, Seongnam, Republic of Korea

[5]Department of Radiology, Seoul National University Bundang Hospital, Seongnam, Republic of Korea

[6]Division of Cardiology, Department of Internal Medicine, Seoul National University Bundang Hospital, Seongnam, Republic of Korea

*These authors contributed equally to this work.

**Supplementary Fig. S1.** Box plot of the squared error values in the four test-sets: normal, hypertension, diabetes, and smoking. ANOVA demonstrated significant difference between four test sets.

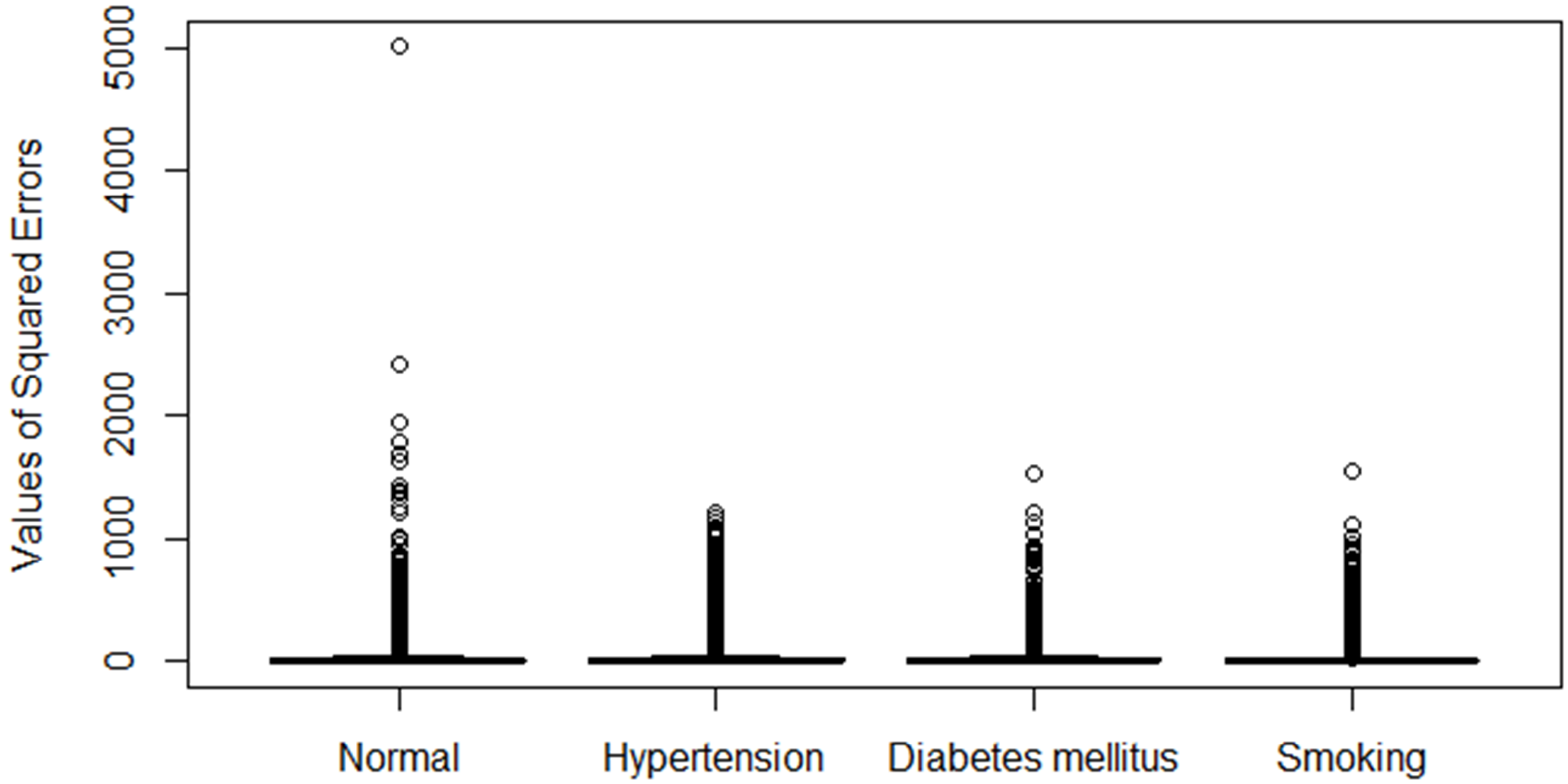

**Supplementary Table S1.** Detailed numbers of participants and their retinal fundus images from both right and left eyes according to underlying conditions.

| | Participants | Images |
|---|---|---|
| Normal | 85,639 | 243,668 |
| Training-set | 84,526 | 216,866 |
| Validation-set | 2,397 | 2,436 |
| Test-set | 20,823 | 24,366 |
| Vascular underlying conditions | | |
| Hypertension | 12,168 | 40,569 |
| Diabetes mellitus | 4,545 | 14,189 |
| Smoking | 30,990 | 113,510 |
| Overlap conditions | | |
| Hypertension & Diabetes mellitus | 2,013 | 7,598 |
| Hypertension & Smoking | 6,500 | 23,165 |
| Diabetes mellitus & Smoking | 2,758 | 9,112 |
| Hypertension & Diabetes mellitus & Smoking | 1,285 | 4,872 |

**Supplementary Table S2.** Number and percentage of retinal fundus images according to the age and sex of each test-set.

| | Normal test-set (N=24,366) | | Hypertension test-set (N=40,569) | | Diabetes mellitus test-set (N=14,189) | | Smoking test-set (N=113,510) | |
|---|---|---|---|---|---|---|---|---|
| Age | Male (N=13,427) | Female (N=10,939) | Male (N=27,389) | Female (N=13,270) | Male (N=10,509) | Female (N=3,680) | Male (N=106,116) | Female (N=7,394) |
| 1 | - | 2 (0.02%) | - | - | - | - | - | - |
| 2 | 2 (0.01%) | 2 (0.02%) | - | - | - | - | - | - |
| 3 | 13 (0.10%) | 25 (0.23%) | - | - | - | - | - | - |
| 4 | 52 (0.39%) | 69 (0.63%) | - | - | - | - | - | - |
| 5 | 109 (0.81%) | 102 (0.93%) | - | - | - | - | - | - |
| 6 | 133 (0.99%) | 133 (1.22%) | - | - | - | - | - | - |
| 7 | 96 (0.71%) | 139 (1.27%) | - | - | - | - | - | - |
| 8 | 84 (0.63%) | 109 (1.00%) | - | - | - | - | - | - |
| 9 | 84 (0.63%) | 76 (0.69%) | - | - | - | - | - | - |
| 10 | 63 (0.47%) | 52 (0.48%) | - | - | - | - | - | - |
| 11 | 47 (0.35%) | 51 (0.47%) | - | - | - | - | - | - |
| 12 | 37 (0.28%) | 41 (0.37%) | - | - | - | - | - | - |
| 13 | 25 (0.19%) | 26 (0.24%) | - | - | - | - | - | - |
| 14 | 26 (0.19%) | 26 (0.24%) | - | - | - | - | - | - |
| 15 | 22 (0.16%) | 18 (0.16%) | - | - | - | - | - | - |
| 16 | 19 (0.14%) | 24 (0.22%) | - | - | - | - | - | - |
| 17 | 14 (0.10%) | 17 (0.16%) | - | - | - | - | - | - |
| 18 | 9 (0.07%) | 11 (0.10%) | - | - | - | - | - | - |
| 19 | 6 (0.04%) | 10 (0.09%) | - | - | - | - | 8 (0.01%) | 2 (0.03%) |
| 20 | 36 (0.27%) | 8 (0.07%) | 4 (0.01%) | - | - | - | 44 (0.04%) | 10 (0.14%) |
| 21 | 36 (0.27%) | 25 (0.23%) | 2 (0.01%) | - | - | 2 (0.05%) | 61 (0.06%) | 10 (0.14%) |
| 22 | 20 (0.15%) | 25 (0.23%) | 6 (0.02%) | - | - | 2 (0.05%) | 72 (0.07%) | 22 (0.30%) |
| 23 | 26 (0.19%) | 21 (0.19%) | 4 (0.01%) | - | 2 (0.02%) | - | 72 (0.07%) | 17 (0.23%) |
| 24 | 23 (0.17%) | 28 (0.26%) | 4 (0.01%) | 2 (0.02%) | - | 4 (0.11%) | 118 (0.11%) | 37 (0.50%) |
| 25 | 29 (0.22%) | 24 (0.22%) | 2 (0.01%) | - | 2 (0.02%) | - | 109 (0.10%) | 32 (0.43%) |
| 26 | 23 (0.17%) | 33 (0.30%) | 12 (0.04%) | 2 (0.02%) | 2 (0.02%) | - | 150 (0.14%) | 49 (0.66%) |
| 27 | 40 (0.30%) | 41 (0.37%) | 10 (0.04%) | - | 2 (0.02%) | - | 201 (0.19%) | 49 (0.66%) |
| 28 | 41 (0.31%) | 46 (0.42%) | 10 (0.04%) | 4 (0.03%) | - | 2 (0.05%) | 240 (0.23%) | 78 (1.05%) |
| 29 | 50 (0.37%) | 66 (0.60%) | 20 (0.07%) | 2 (0.02%) | - | 4 (0.11%) | 320 (0.30%) | 99 (1.34%) |
| 30 | 87 (0.65%) | 62 (0.57%) | 32 (0.12%) | 6 (0.05%) | 4 (0.04%) | - | 489 (0.46%) | 120 (1.62%) |
| 31 | 84 (0.63%) | 84 (0.77%) | 26 (0.09%) | 10 (0.08%) | 6 (0.06%) | 4 (0.11%) | 495 (0.47%) | 114 (1.54%) |
| 32 | 92 (0.69%) | 94 (0.86%) | 28 (0.10%) | - | 4 (0.04%) | 10 (0.27%) | 583 (0.55%) | 140 (1.89%) |
| 33 | 98 (0.73%) | 94 (0.86%) | 50 (0.18%) | 4 (0.03%) | 16 (0.15%) | 10 (0.27%) | 677 (0.64%) | 129 (1.74%) |
| 34 | 125 (0.93%) | 115 (1.05%) | 50 (0.18%) | 10 (0.08%) | 12 (0.11%) | 10 (0.27%) | 830 (0.78%) | 166 (2.25%) |
| 35 | 142 (1.06%) | 125 (1.14%) | 78 (0.28%) | 4 (0.03%) | 12 (0.11%) | 10 (0.27%) | 995 (0.94%) | 182 (2.46%) |
| 36 | 177 (1.32%) | 158 (1.44%) | 84 (0.31%) | 11 (0.08%) | 18 (0.17%) | 22 (0.60%) | 1,274 (1.20%) | 233 (3.15%) |
| 37 | 227 (1.69%) | 185 (1.69%) | 127 (0.46%) | 16 (0.12%) | 46 (0.44%) | 16 (0.43%) | 1,641 (1.55%) | 258 (3.49%) |
| 38 | 215 (1.60%) | 205 (1.87%) | 155 (0.57%) | 18 (0.14%) | 56 (0.53%) | 9 (0.24%) | 1,843 (1.74%) | 279 (3.77%) |
| 39 | 264 (1.97%) | 245 (2.24%) | 189 (0.69%) | 22 (0.17%) | 56 (0.53%) | 23 (0.62%) | 2,007 (1.89%) | 301 (4.07%) |

| | | | | | | | | |
|---|---|---|---|---|---|---|---|---|
| 40 | 299 (2.23%) | 289 (2.64%) | 251 (0.92%) | 48 (0.36%) | 103 (0.98%) | 26 (0.71%) | 2,513 (2.37%) | 300 (4.06%) |
| 41 | 419 (3.12%) | 277 (2.53%) | 346 (1.26%) | 48 (0.36%) | 136 (1.29%) | 26 (0.71%) | 3,127 (2.95%) | 258 (3.49%) |
| 42 | 414 (3.08%) | 293 (2.68%) | 407 (1.49%) | 65 (0.49%) | 157 (1.49%) | 28 (0.76%) | 3,373 (3.18%) | 285 (3.85%) |
| 43 | 484 (3.60%) | 295 (2.70%) | 519 (1.89%) | 94 (0.71%) | 172 (1.64%) | 43 (1.17%) | 3,848 (3.63%) | 308 (4.17%) |
| 44 | 465 (3.46%) | 310 (2.83%) | 556 (2.03%) | 108 (0.81%) | 166 (1.58%) | 35 (0.95%) | 3,713 (3.50%) | 259 (3.50%) |
| 45 | 456 (3.40%) | 295 (2.70%) | 604 (2.21%) | 114 (0.86%) | 200 (1.90%) | 29 (0.79%) | 4,014 (3.78%) | 234 (3.16%) |
| 46 | 472 (3.52%) | 320 (2.93%) | 693 (2.53%) | 104 (0.78%) | 262 (2.49%) | 36 (0.98%) | 4,182 (3.94%) | 255 (3.45%) |
| 47 | 484 (3.60%) | 315 (2.88%) | 711 (2.60%) | 173 (1.30%) | 302 (2.87%) | 50 (1.36%) | 4,215 (3.97%) | 256 (3.46%) |
| 48 | 491 (3.66%) | 319 (2.92%) | 824 (3.01%) | 181 (1.36%) | 357 (3.40%) | 44 (1.20%) | 4,276 (4.03%) | 192 (2.60%) |
| 49 | 489 (3.64%) | 301 (2.75%) | 939 (3.43%) | 222 (1.67%) | 384 (3.65%) | 48 (1.30%) | 4,426 (4.17%) | 231 (3.12%) |
| 50 | 465 (3.46%) | 317 (2.90%) | 931 (3.40%) | 320 (2.41%) | 411 (3.91%) | 61 (1.66%) | 4,221 (3.98%) | 242 (3.27%) |
| 51 | 517 (3.85%) | 339 (3.10%) | 1,200 (4.38%) | 355 (2.68%) | 456 (4.34%) | 63 (1.71%) | 4,970 (4.68%) | 205 (2.77%) |
| 52 | 435 (3.24%) | 343 (3.14%) | 1,058 (3.86%) | 343 (2.58%) | 396 (3.77%) | 82 (2.23%) | 4,085 (3.85%) | 183 (2.47%) |
| 53 | 413 (3.08%) | 316 (2.89%) | 1,124 (4.10%) | 428 (3.23%) | 427 (4.06%) | 89 (2.42%) | 4,107 (3.87%) | 213 (2.88%) |
| 54 | 408 (3.04%) | 319 (2.92%) | 1,165 (4.25%) | 510 (3.84%) | 460 (4.38%) | 90 (2.45%) | 3,986 (3.76%) | 198 (2.68%) |
| 55 | 413 (3.08%) | 262 (2.40%) | 1,283 (4.68%) | 534 (4.02%) | 450 (4.28%) | 102 (2.77%) | 3,893 (3.67%) | 165 (2.23%) |
| 56 | 339 (2.52%) | 245 (2.24%) | 1,104 (4.03%) | 516 (3.89%) | 447 (4.25%) | 97 (2.64%) | 3,380 (3.19%) | 156 (2.11%) |
| 57 | 313 (2.33%) | 230 (2.10%) | 1,112 (4.06%) | 494 (3.72%) | 450 (4.28%) | 108 (2.93%) | 3,153 (2.97%) | 140 (1.89%) |
| 58 | 316 (2.35%) | 242 (2.21%) | 1,114 (4.07%) | 467 (3.52%) | 464 (4.42%) | 149 (4.05%) | 2,934 (2.76%) | 136 (1.84%) |
| 59 | 247 (1.84%) | 232 (2.12%) | 1,058 (3.86%) | 614 (4.63%) | 374 (3.56%) | 171 (4.65%) | 2,464 (2.32%) | 110 (1.49%) |
| 60 | 228 (1.70%) | 203 (1.86%) | 947 (3.46%) | 638 (4.81%) | 368 (3.50%) | 206 (5.60%) | 2,195 (2.07%) | 105 (1.42%) |
| 61 | 214 (1.59%) | 192 (1.76%) | 934 (3.41%) | 591 (4.45%) | 352 (3.35%) | 110 (2.99%) | 2,044 (1.93%) | 65 (0.88%) |
| 62 | 191 (1.42%) | 182 (1.66%) | 718 (2.62%) | 523 (3.94%) | 302 (2.87%) | 159 (4.32%) | 1,732 (1.63%) | 88 (1.19%) |
| 63 | 174 (1.30%) | 185 (1.69%) | 737 (2.69%) | 554 (4.17%) | 279 (2.65%) | 158 (4.29%) | 1,610 (1.52%) | 71 (0.96%) |
| 64 | 148 (1.10%) | 165 (1.51%) | 648 (2.37%) | 538 (4.05%) | 308 (2.93%) | 179 (4.86%) | 1,503 (1.42%) | 55 (0.74%) |
| 65 | 176 (1.31%) | 142 (1.30%) | 675 (2.46%) | 491 (3.70%) | 266 (2.53%) | 149 (4.05%) | 1,415 (1.33%) | 52 (0.70%) |
| 66 | 155 (1.15%) | 145 (1.33%) | 625 (2.28%) | 485 (3.65%) | 246 (2.34%) | 146 (3.97%) | 1,247 (1.18%) | 55 (0.74%) |
| 67 | 118 (0.88%) | 151 (1.38%) | 589 (2.15%) | 395 (2.98%) | 214 (2.04%) | 130 (3.53%) | 1,061 (1.00%) | 34 (0.46%) |
| 68 | 151 (1.12%) | 136 (1.24%) | 530 (1.94%) | 457 (3.44%) | 189 (1.80%) | 137 (3.72%) | 1,030 (0.97%) | 30 (0.41%) |
| 69 | 112 (0.83%) | 105 (0.96%) | 509 (1.86%) | 382 (2.88%) | 156 (1.48%) | 123 (3.34%) | 942 (0.89%) | 29 (0.39%) |
| 70 | 114 (0.85%) | 105 (0.96%) | 439 (1.60%) | 406 (3.06%) | 140 (1.33%) | 126 (3.42%) | 786 (0.74%) | 38 (0.51%) |
| 71 | 84 (0.63%) | 108 (0.99%) | 338 (1.23%) | 312 (2.35%) | 157 (1.49%) | 75 (2.04%) | 642 (0.60%) | 25 (0.34%) |
| 72 | 80 (0.60%) | 68 (0.62%) | 370 (1.35%) | 347 (2.61%) | 155 (1.47%) | 103 (2.80%) | 589 (0.56%) | 18 (0.24%) |
| 73 | 84 (0.63%) | 74 (0.68%) | 286 (1.04%) | 288 (2.17%) | 99 (0.94%) | 78 (2.12%) | 495 (0.47%) | 6 (0.08%) |
| 74 | 76 (0.57%) | 80 (0.73%) | 232 (0.85%) | 208 (1.57%) | 79 (0.75%) | 61 (1.66%) | 396 (0.37%) | 19 (0.26%) |
| 75 | 43 (0.32%) | 58 (0.53%) | 183 (0.67%) | 201 (1.51%) | 79 (0.75%) | 43 (1.17%) | 280 (0.26%) | 14 (0.19%) |
| 76 | 49 (0.36%) | 63 (0.58%) | 174 (0.64%) | 151 (1.14%) | 67 (0.64%) | 53 (1.44%) | 260 (0.25%) | 8 (0.11%) |
| 77 | 38 (0.28%) | 68 (0.62%) | 155 (0.57%) | 130 (0.98%) | 69 (0.66%) | 37 (1.01%) | 215 (0.20%) | 6 (0.08%) |
| 78 | 33 (0.25%) | 56 (0.51%) | 100 (0.37%) | 81 (0.61%) | 29 (0.28%) | 28 (0.76%) | 151 (0.14%) | 8 (0.11%) |
| 79 | 31 (0.23%) | 35 (0.32%) | 85 (0.31%) | 70 (0.53%) | 29 (0.28%) | 28 (0.76%) | 121 (0.11%) | 6 (0.08%) |
| 80 | 19 (0.14%) | 22 (0.20%) | 42 (0.15%) | 58 (0.44%) | 28 (0.27%) | 8 (0.22%) | 74 (0.07%) | 7 (0.09%) |
| 81 | 20 (0.15%) | 23 (0.21%) | 66 (0.24%) | 40 (0.30%) | 35 (0.33%) | 17 (0.46%) | 82 (0.08%) | - |
| 82 | 8 (0.06%) | 23 (0.21%) | 35 (0.13%) | 24 (0.18%) | 20 (0.19%) | 13 (0.35%) | 45 (0.04%) | - |

| | | | | | | | | |
|---|---|---|---|---|---|---|---|---|
| 83 | 18 (0.13%) | 21 (0.19%) | 14 (0.05%) | 25 (0.19%) | 4 (0.04%) | 6 (0.16%) | 34 (0.03%) | 2 (0.03%) |
| 84 | 10 (0.07%) | 10 (0.09%) | 16 (0.06%) | 10 (0.08%) | 7 (0.07%) | - | 20 (0.02%) | - |
| 85 | 13 (0.10%) | 17 (0.16%) | 21 (0.08%) | 9 (0.07%) | 4 (0.04%) | - | 22 (0.02%) | - |
| 86 | 10 (0.07%) | 2 (0.02%) | 14 (0.05%) | - | 6 (0.06%) | - | 5 (0.00%) | - |
| 87 | 3 (0.02%) | 6 (0.05%) | 7 (0.03%) | 7 (0.05%) | 4 (0.04%) | 2 (0.05%) | 11 (0.01%) | - |
| 88 | 4 (0.03%) | 8 (0.07%) | 4 (0.01%) | - | 4 (0.04%) | - | - | - |
| 89 | 1 (0.01%) | 4 (0.04%) | 2 (0.01%) | - | - | - | - | - |
| 90 | 3 (0.02%) | 4 (0.04%) | - | - | - | - | - | - |
| 91 | 2 (0.01%) | 1 (0.01%) | 2 (0.01%) | - | 2 (0.02%) | - | - | - |
| 92 | 1 (0.01%) | - | - | - | - | - | - | - |
| 93 | 1 (0.01%) | - | - | - | - | - | - | - |
| 94 | - | - | - | - | - | - | - | - |
| 95 | - | - | - | - | - | - | - | - |
| 96 | - | 1 (0.01%) | - | - | - | - | - | - |

**Supplementary Table S3.** Statistical Significance of squared error values ([predicted age-chronologic age]$^2$) between test-sets ($P < 0.05$).

| Test-set | Normal | Hypertension | Diabetes mellitus | Smoking |
|---|---|---|---|---|
| Normal | | Yes | Yes | Yes |
| Hypertension | Yes | | Yes | Yes |
| Diabetes mellitus | Yes | Yes | | Yes |
| Smoking | Yes | Yes | Yes | |

Yes = statistical significance between groups (ANOVA, Tukey’s post hoc test, $P < 0.05$).
